# A Comprehensive, Multi-modal Evaluation of the Assessment System of an Undergraduate Research Methodology Course: Translating Theory into Practice

**Published:** 2014

**Authors:** Hamza Mohammad Abdulghani, Gominda G. Ponnamperuma, Farah Ahmad, Zubair Amin

**Affiliations:** 1 Hamza Mohammad Abdulghani, FRCGP, Associate Professor, College of Medicine, King Saud University, P.O. Box: 230155, Riyadh 11321, Kingdom of Saudi Arabia.; 2 Gominda G. Ponnamperuma, PhD, College of Medicine, University of Colombo, Colombo, Sri Lanka; 3 Farah Ahmad, PhD, Department of Medical Education, College of Medicine, King Saud University.; 4 Zubair Amin, MD, Associate Professor, Department of Pediatrics, College of Medicine, National University of Singapore, Singapore.

**Keywords:** Assessment, Evaluation, Utility Criteria, Research Course

## Abstract

***Objective:*** To evaluate assessment system of the 'Research Methodology Course' using utility criteria (i.e. validity, reliability, acceptability, educational impact, and cost-effectiveness). This study demonstrates comprehensive evaluation of assessment system and suggests a framework for similar courses.

***Methods:*** Qualitative and quantitative methods used for evaluation of the course assessment components (50 MCQ, 3 Short Answer Questions (SAQ) and research project) using the utility criteria. Results of multiple evaluation methods for all the assessment components were collected and interpreted together to arrive at holistic judgments, rather than judgments based on individual methods or individual assessment.

***Results:*** Face validity, evaluated using a self-administered questionnaire (response rate-88.7%) disclosed that the students perceived that there was an imbalance in the contents covered by the assessment. This was confirmed by the assessment blueprint. Construct validity was affected by the low correlation between MCQ and SAQ scores (r=0.326). There was a higher correlation between the project and MCQ (r=0.466)/SAQ (r=0.463) scores. Construct validity was also affected by the presence of recall type of MCQs (70%; 35/50), item construction flaws and non-functioning distractors. High discriminating indices (>0.35) were found in MCQs with moderate difficulty indices (0.3-0.7). Reliability of the MCQs was 0.75 which could be improved up to 0.8 by increasing the number of MCQs to at least 70. A positive educational impact was found in the form of the research project assessment driving students to present/publish their work in conferences/peer reviewed journals**. **Cost per student to complete the course was US$164.50.

***Conclusions:*** The multi-modal evaluation of an assessment system is feasible and provides thorough and diagnostic information. Utility of the assessment system could be further improved by modifying the psychometrically inappropriate assessment items.

## INTRODUCTION

Assessment is one of the most important elements that drive students’ learning^1^ and curriculum outcomes.^2^ A good assessment supports students’ learning; whereas a badly constructed and conducted assessment has many negative ramifications including poor grades, de-motivation, curriculum misalignment and disinterest among faculty and students.^2^^,^^3^

Despite the recent calls for evaluation of assessment at the programmatic level^4^, there is sparse evidence for comprehensive evaluation of an assessment system in the literature. A systematic literature review on evaluation of assessment of various courses identified two important shortfalls. First, most are confined to the evaluation of a single parameter (e.g. reliability, validity) of individual assessment instruments (e.g. MCQ, OSCE) rather than the overall assessment system.^5^ Second, data reported are limited to validity (including objectivity) and reliability, but sparse on the impact of assessment on education (i.e. whether the assessment has compelled the students to learn), and feasibility (including cost-effectiveness) and acceptability, despite growing consensus that all these attributes should be taken into account in any evaluation of assessment.^3^^,^^5^ Therefore, we embarked on an action research to ascertain the usefulness and feasibility of comprehensively evaluating the utility of the assessment system of an undergraduate research methodology course through the analysis of both psychometric (validity and reliability) and non-psychometric (educational impact, acceptability, and cost) attributes. As methodology, we chose action research which provides educators an opportunity to engage in deeper exploration to understand the process of teaching and learning in their own contexts.^6^

Therefore this study was initiated to examine the psychometric properties (validity and reliability) of the course assessment and to determine the educational impact, acceptability and cost-effectiveness of the current assessment system of the course. We intend to propose a scheme for comprehensive and multi-modal evaluation of an assessment system that other interested researchers can adopt and adapt.

## METHODS

Undergraduate research methodology is a two-credit course, offered to the 3^rd^ year students longitudinally over one academic year. The aim was ‘to enable students to gain research methodology skills required to plan and carry out a research project, and write a scientific paper using a prescribed format and protocol, under a supervisor in small groups. In addition, there are other teaching and learning topics covered in this course such as epidemiological research, research protocol, biostatistics, and scientific writing. Assessment consists of 50 single best MCQs that carry 40% marks, three assignments in the form of SAQs that carry 25% marks, and a final research project that carries 35% marks.

Validity was established through four criteria^7^; selection of suitable assessment tool(s); suitability and adequacy of curriculum outcomes and contents represented in the assessment material (i.e. content validity); compatibility between the theoretically expected and actual examination results (i.e. construct validity); and fairness (i.e. face validity) of the entire examination system.

A master assessment blueprint was developed against all course objectives to evaluate content validity. Construct validity of the assessment was supported by the correlation between scores of MCQ, SAQ and research project. The hypothesis for correlation between the scores of MCQ and research project, and SAQ and research project was that there may not be a strong positive correlation between each of the two sets of scores, because these two comparisons comprise scores that represent two separate domains of learning, i.e. knowledge (MCQ and SAQ) and skills (research project). However, the correlation between MCQ and SAQ scores was expected to be moderately to highly positive as these two assessments assess the same domain of learning. Further evidence to support construct validity came from the analysis of the level of knowledge assessed in each MCQ, carried out independently by the first and second authors, using a format derived from Blooms Taxonomy^8^. MCQs which assessed only recall and comprehension were classified as K1, and those which assessed application, analysis and evaluation as K2. MCQs were also evaluated for difficulty/discriminating indices, utilization of distractors (by using Question Mark Perception software), and item flaws.^9^^,^^10^

The cut-off values used to evaluate the difficulty index of MCQs were: >0.9 (very easy); 0.9-0.7 (easy); 0.7-0.3 (moderate); 0.3-0.2 (difficult); and <0.2 (very difficult).^11^ Similarly, the cut-off values for discrimination index were: >0.35 (high); 0.35-0.2 (moderate); and <0.2 (poor).^12^

The generalizability theory (G-theory)^13^ was used to determine the reliability of the MCQs, together with the error contribution by different facets (e.g., candidates, exam items), their interactions (e.g., interaction between candidates and exam items), and unsystematic error, to the overall error. Reliability was not estimated for the SAQ and the research project due to insufficient data points. A decision study (D-study) was used to estimate the optimum number of the exam items. G-theory and D-study analyses were conducted using GENOVA for PC software.

The main educational outcome was the completion of the research project. The other desirable but non-mandatory outcome was presentation in conferences or publication in peer-reviewed journals. Whether the course assessment encouraged the students to achieve these outcomes was verified by calculating the number of students who completed the research project, and by surveying the number of students who published their work in scientific forums.

Acceptability of the course among the students (n=248) was established by a questionnaire survey that addressed relevant issues such as coverage of course contents/objectives by the assessment, examiners, and timing of assessment. All items in the questionnaire were rated on a 3-point (agree, true sometimes, and disagree) scale. The results were analyzed by calculating the average rating for each questionnaire item.

A Focus Group Discussion (FGD) was conducted with 10 male and 10 female students to ensure deeper discussion on critical issues identified through the questionnaire. All comments were categorized thematically, using constant-comparative method by investigators.

Since the entire undergraduate program is funded by the government, it was not possible to apportion the real cost of the course assessment. However, the cost was hypothetically determined by calculating the total cost (faculty and student time, and administrative and resource cost) of the assessment and dividing the said total cost by the number of students. The study was approved by the Research Ethical Committee, College of Medicine. Table-I summarizes the methods used to evaluate the course assessment system.

## RESULTS

The total number of the students was 248 (male 143, 57.7%; female 105, 42.3%). The total number of projects was 68. All students passed the course. The response rate for the feedback was 88.7% (248/225).

***Content validity:*** The master blueprint indicated that the majority of the topics [i.e. 17/27 (63%)] were tested by MCQs, while all the objectives (100%) were tested by the project,. The majority of MCQs (29 out of 50; 58%) were context free (K1 type). Conversely, 21 out of 50 MCQs (42%) were context rich (K2 type).

***Construct validity:*** All three assessment scores showed moderate, positive, and significant Pearson’s correlation coefficients (Table-II). 

**Fig.1:**
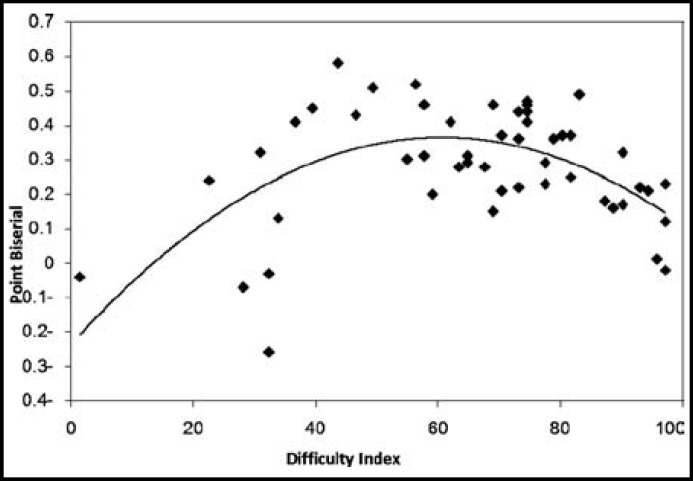
Scatter plot showing the relationship between the difficulty and discrimination indices

Among MCQs with flawed items (n=25; 50%) as identified by the investigators, 11 (22%) had negative questions, and 14 (28%) could not fulfill the cover test. Analysis of MCQs showed that K1 type of MCQs (29/50; 58%) had more item flaws than the K2 type of MCQs (21/50; 42%). The scatter plot (Fig.1) shows the relationship between the difficulty and discriminating indices. High discriminating indices (0.35 or above) were found in MCQs with moderate difficulty indices (0.3-0.7). Poor discriminating indices (<0.2) were found in easy or very easy (>0.7) MCQs.

***Face validity: ***Response rate for the questionnaire was 88.7% (225/248).Students disagreed about the clarity of course outline (61%), course objectives (62.3%), availability of materials (56%), appropriateness of examination system in terms of its relevance to the course and to the students' future practice (51.8%), receiving feedback (66%), and marks allocation (56.4%). The highest positive agreement was that the exam results were announced at the appropriate time (73.2%).

***Focus group discussion:*** Three major themes emerged: (1) heaviness of the assessment contents, (2) overemphasis of biostatistics, and (3) unbalanced scoring system.

***Heaviness of the assessment contents:*** Students expressed that the assessment contents were very heavy and might not be related to the main outcome of the course. A typical response from students was: 

*"Assessment contents, especially, theoretical parts were very heavy; this affected our final results."*

***Overemphasis of biostatistics: ***Students complained about over-representation of theoretical aspects of biostatistics in the MCQ test. This statement encapsulate students’ response: 

*"The MCQ part mainly depends on the biostatistics components, which are difficult to understand. This affected our scores in our final exam."*

*''Most of the topics in biostatistics which were taught in lectures and appeared in our exam did not have any application in doing the research."*

***Unbalanced scoring system: ***Students admitted that three parts of the exam were appropriate but the mark distribution was unfair. They stated that maximum marks should be allotted to the research project**. **Here are typical responses: 

*"Project marking was fair, otherwise all of us will fail but more marks need to be allocated for the project as this is the main purpose of the course."*

*"Assessment should be based only on our research project. The theory part of the course was very difficult to understand and did not have much application in our projects".*

***Reliability:*** The generalizability coefficient for the MCQ was 0.75 with a standard error of measurement of 0.06. The variance components of different facets are shown in Table-III. Variance component for persons (candidates) was the smallest. The variance component for the interaction between persons and items and for unsystematic error was the largest, resulting in a less than ideal ratio (0.75) of true (candidate) score variance to total score variance. The estimated numbers of items necessary to achieve a generalizability coefficient of 0.8 or above, established through D-study, showed that if the quality of the MCQ remains unchanged there should be about 70 items to achieve 0.8 reliability.

***Educational Impact:*** The course assessment system showed positive educational impact. Out of the 68 research projects submitted for assessment by the students, 11 (16%) were accepted for publication in peer reviewed journals and 24 (35%) were accepted at national and international conferences.

***Cost-effectiveness:*** The approximate cost was calculated as 152,956 Saudi Riyals (USD 40,788). This is equivalent to US$164.50/student.

## DISCUSSION

This is one of the first published studies to demonstrate how a comprehensive evaluation of an assessment system through action research could be conducted. 

Content validity is a major determinant of the quality of assessment, could have been enhanced by properly prepared blueprint.^14^ This would have also clarified some of the course outcomes/objectives^15^, about which the students had negative feedback. Difficulty and discriminating indices indicated that moderately difficult questions had better discriminating power than very difficult or very easy questions, similar to published findings.^16^ This confirms one of the basic principles of item response theory, which postulates that the questions that are at the same level of the average candidate’s ability are the most effective.^12^^,^^16^ Difficulty indices were affected by faulty MCQs - a finding that validates prior research, which shows flaws make the questions difficult to answer.^17^ This mainly affects good students and benefits borderline students.^11^

**Table-I:** Summary of the evaluation methods used to evaluate the course assessment system

*Objectives*	*Methods*					
	*Blueprint*	*Survey*	*Statistical & Item Analysis*	*Focus Group Discussion*	*Quality Questions Analysis*	*Other methods* *(Categorization and totaling)*
Validity	x	x	x	x	x	
Reliability-(MCQ)			x			
Educational Impact		x		x		x
Acceptability		x		x		
Cost Effectiveness						x

**Table-II:** Correlation between the scores of three assessment tools

*Comparison*	*Correlation Coefficient*	*Significance (2-tailed)*
MCQ versus Project	0.466	p <0.01
Project versus SAQ	0.463	p <0.01
MCQ versus SAQ	0.326	p <0.01

**Table-III:** Variance components of facets of MCQ scores

*Facet of MCQ*	*Variance component (%)*
p (candidates)	0.011 (4.9 %)
i (items)	0.029 (12.7%)
p x i (candidates x items interaction & unsystematic error)	0.186 (82.4%)

About one-third of distractors were non-functioning, a finding similar to other studies.^10^ With non-functioning distractors the questions become easy and non-discriminating. This may be the reason for the high percentage of 'easy' and 'very easy' questions found.

Although, a reliability coefficient of 0.75 could be argued as acceptable, a coefficient of ≥0.80 is ideal for summative assessment.^2^ Other study has reported higher values like 0.84.^18^ Adequate sampling, using a properly prepared blueprint, in addition to improving validity by minimizing item flaws, improves reliability^19^ and should be an explicit agenda for question development. The decision study in generalizability analysis showed that 70 MCQs are required to achieve a reliability of 0.80. However, this number could be reduced by constructing better quality MCQs with reduced items flaws. This analysis showed how the evaluation of different criteria (e.g. validity and reliability) can be combined and interpreted to improve the assessment system.

Educational impact analysis, an often neglected topic in evaluation of assessment, showed that assessment of the project has motivated the students to achieve beyond the requirements of the course.

Complaints by students about course assessment during the focus group discussions indicated that the students acceptability of the course is not very high. This could be due to the difficulty that the students have in understanding statistics, which is a core component of the research methodology course. Similar findings have been reported elsewhere.^20^

The theoretical estimation of cost was reasonable and comparable to other similar examinations.^21^ However, with regard to research methodology course examination, there was no study in the literature to compare the findings of this study. Hence, this study might provide a yard-stick for future researchers to calculate and compare cost-effectiveness of similar assessment.

***Limitations of the study: ***First, the evidence supporting construct validity could be only considered as partial, since only examinations assessing different and the same domains were compared; i.e. scores of students with different ability levels for any of the three assessments were not compared. Second, the study context is limited only to one institute and one course. Third, feedback from other stakeholders such as examiners and tutors were not taken into account.

## CONCLUSIONS

This study showed how an evaluation of an assessment could be enhanced by the collation of the results of multi-modal evaluation methods including all utility criteria, and matching student perception. This study is also one real life translation of programmatic assessment to practice, a novel idea propagated recently in literature^4^ where evidence to support the utility comes from holistic interpretation of all data. Hence, it is an illustration of translating theory into practice.

## Notes

*Source of Funding:* The study was funded by the College Research Centre, Deanship of Scientific Research, King Saud University, Riyadh, Saudi Arabia.
